# Fabrication of Pb-Containing PtAu Nanoflowers via Galvanic Replacement Method for Electrocatalytical Oxidation of Methanol

**DOI:** 10.3390/molecules29235492

**Published:** 2024-11-21

**Authors:** Zhao Huang, Zhirou Zhang, Long Chao, Xueen Jia

**Affiliations:** 1 Hunan Key Laboratory of Biomedical Nanomaterials and Devices, College of Life Sciences and Chemistry, Hunan University of Technology, Zhuzhou 412007, China; huang1020@hut.edu.cn (Z.H.); dorazhangzr@163.com (Z.Z.); 2 Key Laboratory of Chemical Biology and Traditional Chinese Medicine Research (Ministry of Education of China), College of Chemistry and Chemical Engineering, Hunan Normal University, Changsha 410081, China; 3 Department of Physics, Umeå University, SE-901 87 Umeå, Sweden

**Keywords:** double-cabin galvanic cell, underpotential deposition, Pb-containing PtAu nanoflower, electrocatalytical oxidation of methanol

## Abstract

A Pb-containing PtAu nanoflower electrocatalyst was deposited on the cathode via galvanic replacement reaction in a double-cabin galvanic cell (DCGC) with a Cu plate as the anode, a multiwalled carbon nanotube (MWCNT) modified glassy carbon electrode (GCE) as the cathode, 0.1 M HClO_4_ aqueous solution as the anolyte, and Pb^2+^-containing Pt^4+^ salt and Au^3+^ salt mixed aqueous solution as the catholyte, respectively, and the electrocatalytic performance of the modified electrode toward methanol oxidation in the alkaline medium was investigated. Electrochemical studies reveal that the stripping of bulk Cu can induce underpotential deposition (UPD) of Pb on Pt during the galvanic replacement reaction, which affects the morphology and composition of Pb-containing PtAu nanoparticles. Under the optimal experimental conditions, a Pb-Pt_3_Au_1_/MWCNTs/GCE shows the highest activity and the best stability toward electrocatalytic oxidation of methanol in the alkaline medium, and the Pt active area-normalized specific electrocatalytic activity of Pb-Pt_3_Au_1_/MWCNTs/GCE is as high as 59.8 mA cm_Pt_^−2^. We believe that the method presented here of depositing highly active noble metal nanostructures by galvanic replacement reaction in a DCGC device is expected to be widely applied in the preparation of nanomaterials for their study in fuel cells and electrocatalysis.

## 1. Introduction

Direct methanol fuel cells (DMFCs) have attracted great interest as a renewable energy supply due to their advantages of low operating temperature, easy operation, and high energy density [1]. Currently, Pt, as a commonly used methanol electrocatalyst, is widely used in DMFC research, but Pt is expensive, scarce, and easily deactivated by CO_ads_ intermediates generated by methanol electrooxidation, which limits its commercial application in DMFCs [2]. Therefore, the key to the development of DMFCs is to develop efficient, low-cost, and anti-poisoning methanol electrocatalysts. Bi (or tri)-component Pt-based electrocatalysts formed by Pt with other transition metals or noble metals can effectively reduce the Pt content and improve the activity and anti-poisoning ability of Pt. So far, various Pt-based multi-component electrocatalysts have been studied, such as Pt-Mn [3], Pt-Fe [4], Pt-Co [5], Pt-Ru [6], Pt-Ni [7], Pt-Cu [8], Pt-Pd [9], Pt-Ag [10], Pt-Mo [11], and PtRuFe [12]. These multi-component Pt-based electrocatalysts can significantly improve the electrocatalytic activity and stability of Pt through electronic effects and bifunctional effects between components.

As a common noble metal, Au exhibits good chemical stability and weak catalytic activity in its bulk state, but nanosized Au particles show excellent electrocatalytic performance for small molecules [13,14]. The bi-component PtAu electrocatalyst can effectively enhance the anti-poisoning capability and catalytic activity of Pt due to the synergistic effect between Pt and Au components [15]. Currently, people use various methods to prepare PtAu electrocatalysts with different morphologies and compositions. For example, Zhang et al. prepared nanoporous PtAu electrocatalysts via dealloying and found that they could efficiently electrocatalyze the oxidation of methanol under acidic conditions [16]. Luo et al. synthesized PtAu nanoparticles supported on carbon via colloidal synthesis and observed that the bifunctional effect of Au could enhance the electrocatalytic activity of Pt toward methanol under alkaline conditions [17]. In addition, doping the PtAu electrocatalyst with other base metals (such as Cu and Pb) can further enhance the catalytic performance of the PtAu-based tri-component electrocatalyst. For instance, Wang et al. synthesized Au-Cu-X (X = Pt, Pd, Ag) ternary metal nanorods using Au-Cu nanorods as a galvanic cell displacement template, and the synthesized nanorods were found to be highly effective in the reduction of 4-nitrophenol [18]. To date, extensive studies have been conducted on the preparation of PtAu electrocatalysts; previous synthesis methods also have disadvantages such as high cost, complex operations, and environmental pollution. The galvanic cell method, due to its low energy consumption, simplicity, and low cost, is a typical green preparation technology. For example, Yoshii et al. synthesized Pd-Co bimetallic nanocatalysts supported on C using Co nanoparticles supported on C as a displacement template and found that they had high catalytic activity and selectivity for the hydrogenation of styrene [19]. In the previous galvanic cell method for the synthesis of Pt-based electrocatalysts, the anode and cathode reactions of the galvanic cell occurred in the same solution. Both reactions at the anode and cathode of the galvanic cell may affect the composition of the deposited Pt-based electrocatalyst. In some cases, further purification of the product is inevitably required, which adds complexity to the process and increases production costs, both of which are undesirable. The use of a double-cabin galvanic cell (DCGC) with separate anode and cathode compartments allows for the one-step deposition of clean, multi-component Pt-based electrocatalysts at the cathode, simplifying the operational steps. To our knowledge, there are rarely reports on the direct preparation of high-activity PtAu-based tri-component nanoelectrocatalyst-modified electrodes at the cathode using the DCGC setup.

In this work, using a high-purity Cu plate as the anode and a glassy carbon electrode (GCE) modified with multiwalled carbon nanotubes (MWCNTs) as the cathode in the DCGC device, a Pb-containing PtAu nanoflower electrocatalyst was deposited at the cathode through galvanic replacement, and the electrocatalytic performance of the resulting modified electrode toward methanol electrooxidation in an alkaline environment was investigated. Electrochemical studies show that the dissolution of bulk Cu during the galvanic reaction can induce underpotential deposition (UPD) of Pb on the Pt surface, leading to the deposition of Pb-containing PtAu nanoflower electrocatalysts on MWCNTs. Pb-containing PtAu nanoflower electrocatalysts exhibit excellent activity toward methanol electrocatalytic oxidation and have a potential application value in DMFCs.

## 2. Results and Discussion

### 2.1. Fabrication and Characterization of Pb-Containing PtAu Nanoparticles

**Figure 1:**
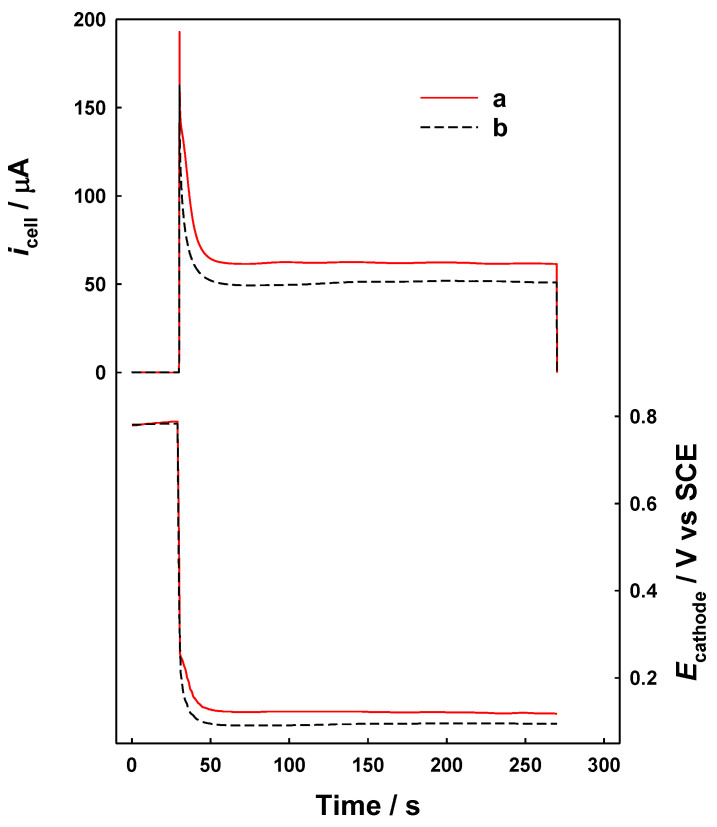
Simultaneous responses of *i*_cell_ and *E*_cathode_ in the DCGC with a Cu plate as the anode, 0.1 M HClO_4_ as the anolyte (stationary), and the MWCNTs/GCE as the cathode, while the catholyte (stationary) was 3.0 mM H_2_PtCl_6_ + 1.0 mM HAuCl_4_ + 5.0 mM Pb(ClO_4_)_2_ + 0.1 M HClO_4_ aqueous solution (a), for the deposition of Pb-Pt_3_Au_1_) and 3.0 mM H_2_PtCl_6_ + 1.0 mM HAuCl_4_ + 0.1 M HClO_4_ aqueous solution (b), for deposition of Pt_3_Au_1_), respectively. Short-circuit discharging of the DCGC (connecting the anode and cathode by a conducting Cu wire) was from 30 to 270 s.

Figure 1 shows the discharging current (*i*_cell_) and the potential of a MWCNT/GCE cathode (E_cathode_) during the depositing of Pb-Pt_3_Au_1_ and Pt_3_Au_1_ on its surface. When the anode and cathode are not connected, the electrochemical potential (ECP) of the Cu plate is approximately 0.008 V in 0.1 M HClO_4_ solution (anode part), while the ECP of the MWCNT/GCE surface in the mixed solution of noble metal salts is about 0.78 V (cathode part). Therefore, after connecting the anode and cathode of the DCGC device with a Cu wire, the ECP difference between the anode and cathode drives the galvanic replacement reaction, leading to the dissolution of bulk Cu (Cu_bulk_) at the anode and the deposition of noble metal nanoparticles on the surface of the MWCNTs/GCE at the cathode. During the deposition of Pt_3_Au_1_ from 3.0 mM H_2_PtCl_6_ + 1.0 mM HAuCl_4_ + 0.1 M HClO_4_ catholyte (without the presence of Pb(ClO_4_)_2_; Figure 1 b), the steady-state potential and *i*_cell_ of MWCNTs/GCE are 0.097 V and 52 μA, respectively. However, during the deposition of Pb-Pt_3_Au_1_ from 3.0 mM H_2_PtCl_6_ + 1.0 mM HAuCl_4_ + 5.0 mM Pb(ClO_4_)_2_ + 0.1 M HClO_4_ (with the presence of Pb(ClO_4_)_2_; Figure 1 a), the steady-state potential and *i*_cell_ of MWCNTs/GCE increase to 0.121 V and 62 μA, respectively. The response for the deposition of Pb-Pt_3_Au_1_ is larger than that for the deposition of Pt_3_Au_1_, which perhaps suggests that the addition of Pb(ClO_4_)_2_ to the catholyte facilitates the deposition of Pt and Au.

**Figure 2:**
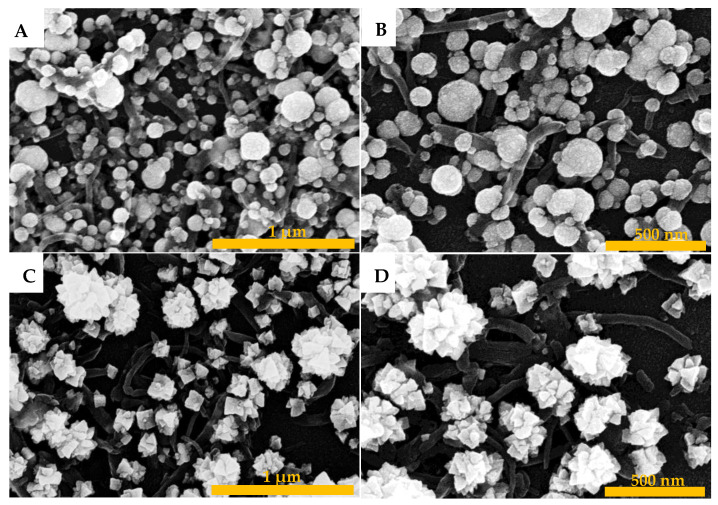
SEM of Pt_3_Au_1_/MWCNTs/GCE (**A**,**B**) and Pb-Pt_3_Au_1_/MWCNTs/GCE (**C**,**D**).

**Figure 3:**
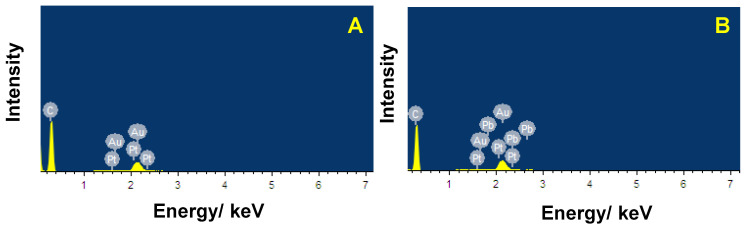
EDS spectra of Pt_3_Au_1_/MWCNTs/GCE (**A**) and Pb-Pt_3_Au_1_/MWCNTs/GCE (**B**), respectively.

Figure 2 shows the SEM images of the obtained Pt_3_Au_1_/MWCNTs/GCE and Pb-Pt_3_Au_1_/MWCNTs/GCE. For Pt_3_Au_1_/MWCNTs/GCE (Figure 2A,B), a large number of spherical nanoparticles with diameters of 40–240 nm are distributed on the surface of MWCNTs. However, in Pb-Pt_3_Au_1_/MWCNTs/GCE (Figure 2C,D), flower-like nanoparticles grow on the surface of MWCNTs, with diameters ranging from 110 to 450 nm, and the density of nanoparticles on the surface of Pb-Pt_3_Au_1_/MWCNTs/GCE is also lower than that of Pt_3_Au_1_/MWCNTs/GCE. EDS characterization (Figure 3) also proved that Pt and Au have been deposited on the surface of MWCNTs/GCE, and the atomic percentages of Pt and Au for Pt_3_Au_1_ nanoparticles were determined to be 53.9% and 46.1% (Figure 3A), respectively, which was unproportional to that of the catholyte solution (mole concentration ratio for *c*_H2PtCl6_:*c*_HAuCl4_ = 3:1). Due to the higher ECP of Au versus that of Pt, the reaction kinetics for reducing AuCl_4_^−^ is anticipated to be significantly faster than that for reducing PtCl_6_^2−^, thus leading to the deposition of more Au in Pt_3_Au_1_ nanoparticles even at a high Pt/Au mole ratio in the catholyte. For Pb-Pt_3_Au_1_/MWCNTs/GCE, there are a small number of Pb atoms present in the obtained PtAu nanoparticles (Figure 3B), and the atomic percentages of Pt, Au, and Pb were determined to be 50.0%, 42.9%, and 7.1%, respectively, which indicated the occurrence of cathodic deposition of Pb from the Pb(ClO_4_)_2_-containing catholyte during the GRR deposition of Pb-Pt_3_Au_1_ nanoparticles, despite the higher activity of bulk lead (Pb_bulk_) compared to Cu_bulk_ (anode here). The above results indicate that when Pb(ClO_4_)_2_ is added to the catholyte, the co-deposition of Pb on the cathode can influence the morphology and composition of PtAu nanoparticles, leading to the formation of Pb-containing PtAu nanoflower particles. Indeed, previous studies have also reported that the addition of transition metal ions (such as Cu^2+^) can control the crystal structure and morphology for synthesizing noble metal nanostructures [20].

Moreover, Figure S1 shows the X-ray diffraction (XRD) patterns of the as-synthesized Pt_3_Au_1_ and Pb-Pt_3_Au_1_ thin film sample on MWCNTs. The peaks at 37.1° and 42.4° are assigned to Au(111) and Pt(111), respectively, according to JCPDS 04-0784 of pure Pt and JCPDS 04-0802 of pure Au, and the peaks at 73.3° and 76.4° may correspond to (311) and (222) planes. In our case, no XRD peaks of Pb are observed on Pb-Pt_3_Au_1_ that may be the Pb of UPD is too small to produce an X-ray diffraction signal. X-ray photoelectron spectroscopy (XPS) was applied to characterize the Pt_3_Au_1_ and Pb-Pt_3_Au_1_ thin film sample also (as shown in Figure S2). The two 4f binding states of zero valence, at 84.4 (4f_5/2_) and 87.5 eV (4f_7/2_) for Au^0^ and at 71.2 (4f_5/2_) and 74.5 eV (4f_7/2_) for Pt^0^, were observed on both Pt_3_Au_1_ and Pb-Pt_3_Au_1_, respectively. Moreover, Pb may exist as Pb^2+^ in Pb-Pt_3_Au_1_, with the peak of Pb^2+^ 4f_7/2_ and 4f_5/2_ appearing at 139.6 eV and 144.6 eV, respectively, which is a large shift compared to pure Pb^0^, indicating the electronic interaction between PtAu and Pb.

### 2.2. Electrochemical Study of the Mechanism of Pb Co-Deposition at the Cathode During the GRR Process in the DCGC Device

**Figure 4:**
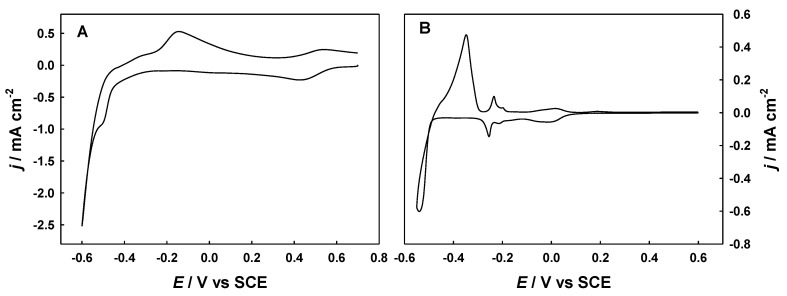
Cyclic voltammograms of Pt_pla_/Au (**A**) and bare Au (**B**) in 3.0 mM Pb(ClO_4_)_2_ + 0.1 M HClO_4_ aqueous solution, respectively. Scan rate: 20 mV s^−1^.

It is significant and intriguing to study the mechanism of Pb co-deposition at the cathode when depositing Pt and Au in the DCGC device with a Cu plate as the anode. Given that the ECP of Pb_bulk_ is lower than that of Cu_bulk_, the dissolution of Cu_bulk_, inducing the deposition of Pb_bulk_ during the GRR for depositing Pb-Pt_3_Au_1_ nanoparticles at the cathode, is thermodynamically impossible. Here, we studied the electrochemical deposition behavior of Pb on Pt or Au surfaces in cyclic voltammetry (CV) experiments. Figure 4 shows the CV curves for electroplated Pt-modified Au (Pt_pla_/Au; as shown in Figure S3) and bare Au electrodes in a solution containing 3.0 mM Pb(ClO_4_)_2_ + 0.1 M HClO_4_. For Pt_pla_/Au, during the negative-going scan, the UPD of Pb on the Pt surface starts at 0.55 V [21]. The UPD of Pb on the Pt surface (Pb^0^_UPD_) significantly impedes the adsorption/desorption behavior of H on the Pt surface between 0 and −0.28 V. The UPD of Pb terminates at −0.45 V, where the bulk deposition of Pb (Pb^0^_bulk_) begins. For the bare Au electrode, during the negative-going scan, the UPD of Pb on Au starts at 0.06 V, and two distinct pairs of oxidation-reduction peaks appear at 0.01/−0.01 V and −0.23/−0.25 V, corresponding to the UPD behavior of Pb on the Au surface [22]. The UPD of Pb terminates at −0.45 V, where the bulk deposition of Pb occurs, and the dissolution peak of bulk Pb appears at −0.35 V. The CV experiments indicate that the deposition/dissolution potentials of Pb^0^_UPD_ are higher than those of Pb^0^_bulk_, i.e., the ECP of Pb^2+^/Pb^0^_UPD_ is higher than that of Pb^2+^/Pb^0^_bulk_, suggesting the thermodynamic possibility of the bulk dissolution of an active metal inducing the UPD of its ions (or other ions) on a relatively less active metal surface (such as Pt or Au).

In the DCGC device with a Cu plate as the anode, during the GRR for depositing Pb-Pt_3_Au_1_, the steady-state potential of the MWCNTs/GCE is 0.121 V, which falls within the potential range for the UPD of Pb on the Pt surface (0.55~−0.45 V) but is higher than the potential range for the UPD of Pb on the Au surface (0.06~−0.45 V). This indicates that during the deposition of Pb-Pt_3_Au_1_, Pb can undergo UPD on the Pt surface but not on the Au surface. Hence, the bulk dissolution of Cu at the anode can induce the UPD of Pb on Pt at the cathode in the DCGC device. The UPD effect of Pb on the Pt surface influences the growth process of PtAu nanoparticles, leading to the deposition of nanoflower structures on the MWCNT surface. Simultaneously, a small number of Pb^0^_UPD_ atoms are embedded within the PtAu nanoparticles, forming a Pb-containing PtAu nanoflower structure.

### 2.3. Electrocatalytical Oxidation of Methanol on Pb-Containing PtAu Nanoparticles in Alkaline Solution

**Figure 5:**
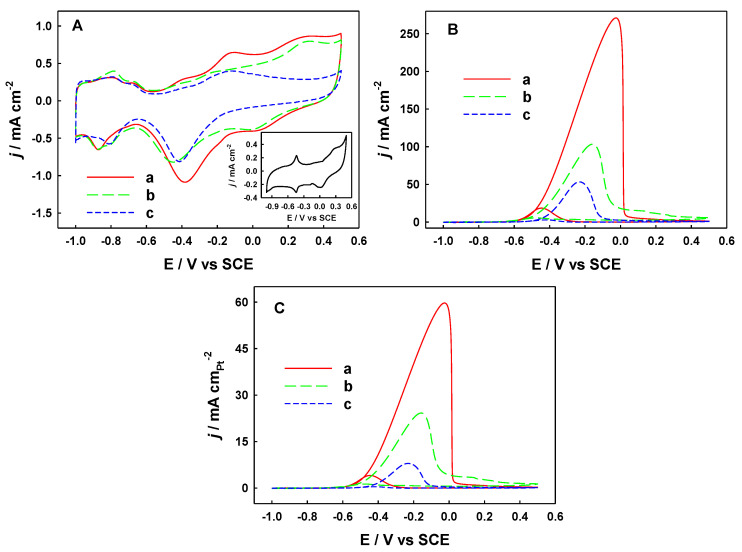
Cyclic voltammograms of Pb-Pt_3_Au_1_/MWCNTs/GCE (a), Pt_3_Au_1_/MWCNTs/GCE (b), and Pb-Pt/MWCNTs/GCE (c) in 1.0 M KOH aqueous solution (**A**) and 1.0 M CH_3_OH + 1.0 M KOH aqueous solution (**B**,**C**), respectively. Currents in plans (**A**–**C**) are normalized to geometric area (**A**,**B**) and Pt active area (**C**), respectively. The inset in plan (**A**) is the CV of Pb-Au/MWCNTs/GCE in 1.0 M KOH aqueous solution. Scan rate: 50 mV s^−1^.

Figure 5 shows the cyclic voltammograms (CVs) of Pb-Pt_3_Au_1_/MWCNTs/GCE, Pt_3_Au_1_/MWCNTs/GCE, and Pb-Pt/MWCNTs/GCE in a 1.0 M KOH (Figure 5A) and 1.0 M CH_3_OH + 1.0 M KOH solution (Figure 5B,C). For Pb-Pt_3_Au_1_/MWCNTs/GCE (Figure 5A), the adsorption/desorption peaks of hydrogen on the Pt surface appear between −0.65 V and −1.0 V. The redox peaks at −0.13 V and −0.38 V correspond to the formation and reduction of Pt oxide [23]. Additionally, the redox peaks at 0.3 V and 0.03 V are attributed to the formation and reduction of Au oxide, which is consistent with the redox peaks of Au on the surface of Pb-Au/MWCNTs/GCE (inset of Figure 5A). The electrochemical characteristic peaks on the surfaces of Pt_3_Au_1_/MWCNTs/GCE and Pb-Pt/MWCNTs/GCE are similar to those of Pb-Pt_3_Au_1_/MWCNTs/GCE.

For the electrocatalytic oxidation of methanol (Figure 5B), during the forward scan, the initial oxidation potential of methanol on the surface of Pb-Pt_3_Au_1_/MWCNTs/GCE is −0.64 V, which is shifted negatively by 30 mV and 130 mV compared to Pt_3_Au_1_/MWCNTs/GCE (−0.61 V) and Pb-Pt/MWCNTs/GCE (−0.51 V), respectively. The peak current for the oxidation of methanol on the surface of Pb-Pt_3_Au_1_/MWCNTs/GCE is 270 mA cm^−2^, which is 2.62 times and 5.0 times higher than that of Pt_3_Au_1_/MWCNTs/GCE (103 mA cm^−2^) and Pb-Pt/MWCNTs/GCE (54 mA cm^−2^), respectively. Furthermore, we compared the electrochemical active area specific activity of Pt (mA cm_Pt_^−2^), as shown in Figure 5C. The peak current for the oxidation of methanol on the surface of Pb-Pt_3_Au_1_/MWCNTs/GCE is 59.8 mA cm_Pt_^−2^, which is 2.48 times and 7.29 times higher than that of Pt_3_Au_1_/MWCNTs/GCE (24.1 mA cm_Pt_^−2^) and Pb-Pt/MWCNTs/GCE (8.2 mA cm_Pt_^−2^), respectively.

The above results indicate that Pb-Pt_3_Au_1_/MWCNTs/GCE exhibits excellent electrocatalytic performance toward methanol, primarily due to the following reasons: (1) compared to the spherical structure of Pt_3_Au_1_ nanoparticles, the Pb-Pt_3_Au_1_ nanoflower particles have more surface defect sites and a higher number of exposed unsaturated Pt atoms, which enhance their catalytic activity [24]; (2) both the bifunctional effect between Pt and Au atoms [17] and the electronic effect between Pt and Pb atoms [25] contribute to enhancing the catalytic performance of the Pb-Pt_3_Au_1_ ternary metal catalyst.

**Figure 6:**
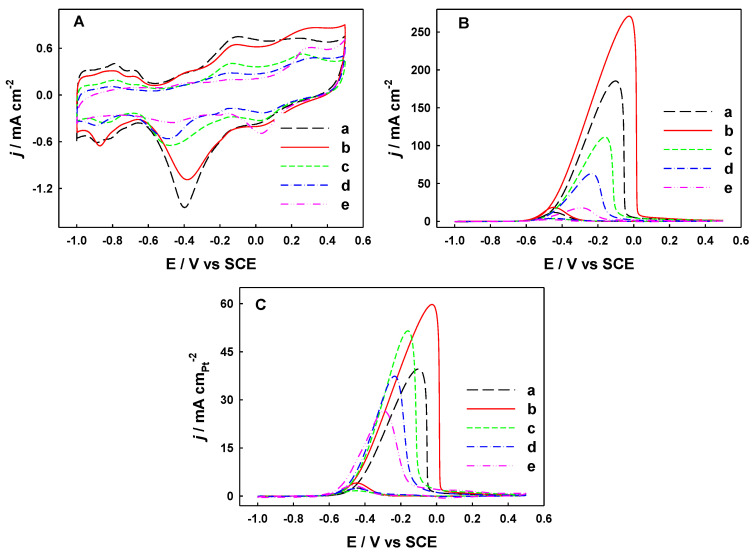
Cyclic voltammograms of Pb-Pt_3_Au_0.5_/MWCNTs/GCE (a), Pb-Pt_3_Au_1_/MWCNTs/GCE (b), Pb-Pt_3_Au_3_/MWCNTs/GCE (c), Pb-Pt_3_Au_5_/MWCNTs/GCE (d), and Pb-Pt_3_Au_7_/MWCNTs/GCE (e) in 1.0 M KOH aqueous solution (**A**) and 1.0 M CH_3_OH + 1.0 M KOH aqueous solution (**B**,**C**), respectively. Currents in plans (**A**–**C**) are normalized to geometric area (**A**,**B**) and Pt active area (**C**), respectively. Scan rate: 50 mV s^−1^.

Since the catalytic performance of noble metal electrocatalysts is related to their morphology, particle size, and composition [26], the modified electrodes with different Pt and Au contents were prepared by changing the concentration ratio of H_2_PtCl_6_ and HAuCl_4_ in the catholyte, and the electrocatalytic performance of the obtained modified electrodes for methanol was studied, as shown in Figure 6. Increasing the concentration of HAuCl_4_ in the catholyte results in an increase in the Au content on the electrode surface, accompanied by a decrease in Pt content, as evidenced by the increased oxidation-reduction peak of Au and the decreased oxidation-reduction peak of Pt (Figure 6A). The peak current density of methanol electrocatalytic oxidation also increases with the increased concentration of HAuCl_4_, reaching a maximum value of 270 mA cm^−2^ at *c*_H2PtCl6_: *c*_HAuCl4_ = 3:1 (Pb-Pt_3_Au_1_/MWCNTs/GCE; Figure 6B), and then decreases with the increased HAuCl_4_ concentration. The electrochemical active area specific activity of Pt (Figure 6C) also reaches a maximum value of 59.8 mA cm_Pt_^−2^ on Pb-Pt_3_Au_1_/MWCNTs/GCE. As the Au content on the electrode surface increases, the synergistic effect between Pt and Au atoms gradually intensifies. The activity of Pt atoms reaches its maximum at a ratio of *c*_H2PtCl6_: *c*_HAuCl4_ = 3:1. As the Au content on the electrode surface continues to increase, the Pt content gradually decreases, as evidenced by the reduced oxidation-reduction peak of Pt observed in the above electrochemical characterization. The probability of Pt atoms being surrounded by Au atoms increases, and some Pt atoms may be separated by Au atoms, reducing the number of adjacent Pt atoms, which may hinder the adsorption and dissociation of methanol on the Pt surface and lead to a decrease in the activity of Pt atoms [27]. It has also been reported that methanol can only undergo electrooxidation on a surface with at least three adjacent Pt atoms [28].

**Figure 7:**
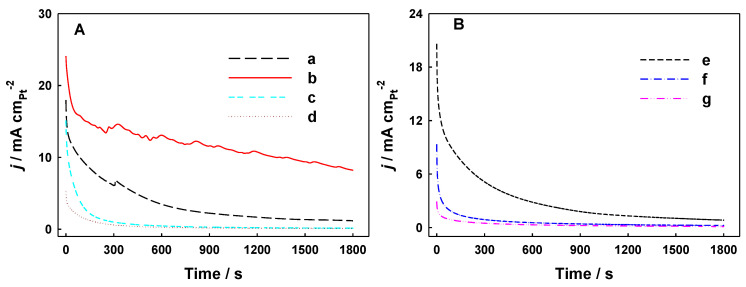
The *i*-t curves at −0.25 V of Pb-Pt_3_Au_0.5_/MWCNTs/GCE (a), Pb-Pt_3_Au_1_/MWCNTs/GCE (b), Pt_3_Au_1_/MWCNTs/GCE (c), Pb-Pt/MWCNTs/GCE (d) (**A**) and Pb-Pt_3_Au_3_/MWCNTs/GCE (e), Pb-Pt_3_Au_5_/MWCNTs/GCE (f), and Pb-Pt_3_Au_7_/MWCNTs/GCE (g) (**B**) in 1.0 M CH_3_OH + 1.0 M KOH aqueous solution, respectively. Currents are normalized to Pt active area.

The electrochemical stability of electrocatalysts is an important indicator for practical applications. We also used a potentiostatic method to evaluate the stability of different modified electrodes in catalyzing the oxidation of methanol in 1.0 M CH_3_OH + 1.0 M KOH solution at −0.25 V, as shown in Figure 7. The Pb-Pt_3_Au_1_/MWCNTs/GCE exhibited the slowest rate of catalytic current decay, with a catalytic current of 8.19 mA cm_Pt_^−2^ at 1800 s, which is significantly higher than that of Pb-Pt/MWCNTs/GCE (0.10 mA cm_Pt_^−2^), Pb-Pt_3_Au_3_/MWCNTs/GCE (0.83 mA cm_Pt_^−2^), Pb-Pt_3_Au_5_/MWCNTs/GCE (0.23 mA cm_Pt_^−2^), and Pb-Pt_3_Au_7_/MWCNTs/GCE (0.11 mA cm_Pt_^−2^). This indicates that the Pb-Pt_3_Au_1_ nanoelectrocatalyst can effectively remove toxic products such as CO_ads_ that are generated during the electrooxidation of methanol, maintaining the catalytic activity and electrochemical stability.

## 3. Materials and Methods

### 3.1. Instrumentation and Reagents

All electrochemical experiments were conducted on an Autolab PGSTAT 30 electrochemical workstation (Eco Chemie BV, Utrecht, The Netherlands) using a conventional three-electrode system. The working electrodes were either a glassy carbon electrode (GCE) with a diameter of 3 mm or a gold electrode (0.29 cm^2^). The reference electrode was a saturated calomel electrode (SCE) equipped with a salt bridge, and the counter electrode was a carbon rod. All potentials were referenced to the SCE. The JEOL JSM-1230 high-resolution field-emission scanning electron microscope (SEM, Akishima, Japan) and the accompanying energy-dispersive X-ray spectroscopy (EDS) were used for morphological characterization and elemental composition analysis, respectively. X-ray diffraction (XRD) pattern was collected on a D8 Discover X-ray diffractometer (Bruker Co., Billerica, MA, USA). X-ray photoelectron spectroscopy (XPS) spectra were taken using an ESCA Escalab 220i XL (Thermo Fisher Scientific, Waltham, MA, USA) with a monochromated Al Kα X-ray source (1486.6 eV).

For the experiments conducted in the DCGC, when no external load was applied, the discharge current (*i*_cell_) of the galvanic cell was monitored using the ECN module of the Autolab PGSTAT 30 electrochemical workstation [29] (Metrohm, Herisau, Switzerland). The accuracy of this setup was verified using a calibrated 1.5 V dry cell (error within 1 mV) under a 100 kΩ load; the dry cell voltage measured by the ECN setup was 1.501 V, with an *i*_cell_ of 15.01 µA, strictly adhering to Ohm’s Law. The potentials of the anode and cathode in the galvanic cell setup were dynamically monitored using a high-impedance (*R* ≥ 10^12^ Ω) voltmeter, which was modified from a pH meter (Leici, Shanghai, China). All experiments were conducted at room temperature (26 ± 2 °C).

*N*,*N*-Dimethylformamide (DMF), HAuCl_4_, H_2_PtCl_6_, and methanol were purchased from Tianjin Chemical Reagent Station (Tianjin, China). Pb(ClO_4_)_2_ and high-purity Cu plate (99.999%) were purchased from Alfa Aesar company (Tianjin, China). Multiwalled carbon nanotubes (MWCNTs; 60 nm outer diameter and 40 nm inner diameter on average) were purchased from Chengdu Organic Chemicals Co. Ltd. (Chengdu, China) and purified in concentrated acids before use. Other reagents were of analytical grade. All solutions were prepared using Milli-Q ultrapure water (Millipore, Burlington, MA, USA, >18 M cm).

### 3.2. Deposition of Pb-Containing PtAu Nanoparticles on MWCNTs/GCE in the DCGC Device

The GCE was sequentially polished with 1.0 μm and 0.05 μm alumina slurry on a chamois leather until a mirror-like finish was achieved. After each polishing step, the surface contaminants were washed off, and the electrode was cleaned in an ultrasonic water bath for 3 min each time; this process was repeated three times. Finally, the electrode was ultrasonically cleaned sequentially with 1:1 ethanol, 1:1 HNO_3_, and distilled water. It was then subjected to cyclic voltammetry (CV) scans in 0.2 M HClO_4_ solution (0 to 1.5 V, 30 mV s^−1^) until reproducible voltammetric responses were observed. The resulting GCE was further subjected to CV scans in 2.0 mM K_4_Fe(CN)_6_ + 0.1 M Na_2_SO_4_ solution (−0.1 to 0.5 V, 50 mV s^−1^). A peak-to-peak potential width of ca. 70 mV indicated that the electrode surface was clean. A 1 mg mL^−1^ dispersion of MWCNTs in DMF was prepared and sonicated for 15 min. Then, 5 μL of the MWCNT dispersion was cast onto the GCE and air-dried to form the MWCNTs/GCE.

**Scheme 1:**
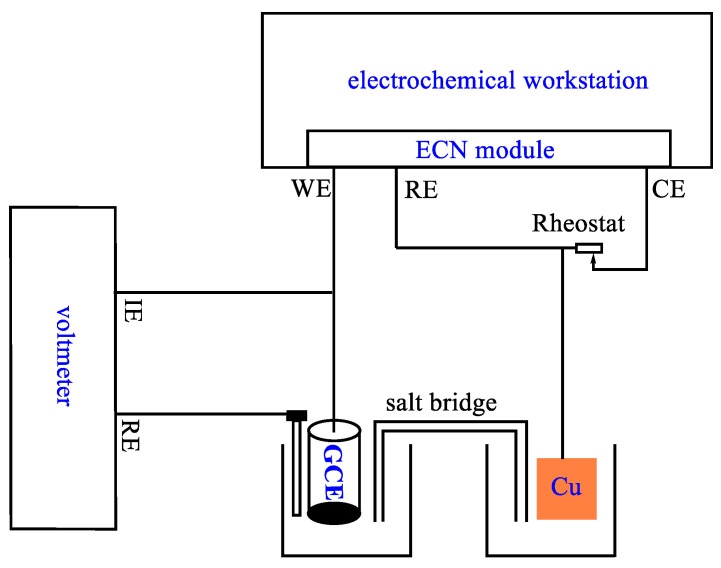
Schematic diagram of the total experimental setup. GCE: glassy carbon electrode. IE: indicator electrode.

For the deposition of Pb-containing PtAu nanoparticles on MWCNTs/GCE in a DCGC device based on the GRR principle, a Cu plate served as the anode, the anolyte was 0.1 M HClO_4_ aqueous solution, MWCNTs/GCE served as the cathode, and the catholyte was 3.0 mM H_2_PtCl_6_ + 1.0 mM HAuCl_4_ + 5.0 mM Pb(ClO_4_)_2_ + 0.1 M HClO_4_ aqueous solution. The catholyte and anolyte were connected using a homemade U-shaped glass tube salt bridge filled with saturated KCl solution. Scheme 1 shows the schematic diagram of the total experimental setup, similar to that of a previous report [30]. After switching on the DCGC, the ECN and voltmeter were used to monitor the *i*_cell_ and the potential of MWCNTs/GCE (vs SCE), respectively. The total discharging time in the DCGC was 240 s. The resulting modified electrode was denoted as Pb-Pt_3_Au_1_/MWCNTs/GCE. Varying the concentration ratio of H_2_PtCl_6_ and HAuCl_4_ in the catholyte can obtain modified electrodes with different Pt and Au content.

For comparative studies, we also prepared other modified electrodes in a similar procedure, and the abbreviation of the modified electrodes and the corresponding composition of catholytes are listed in Table 1.

### 3.3. CV Experiment to Study the Electrochemical Deposition Behavior of Pb on Pt and Au Surfaces

The pretreatment process of the gold electrode was as follows. To remove contaminants from the electrode surface, the gold electrode was first cleaned with H_2_SO_4_ + H_2_O_2_ (*v*/*v*, 3:1) solution for 3 min and then subjected to CV scanning in 0.2 M HClO_4_ solution (0~1.5 V, 30 mV s^−1^) until a reproducible voltammetric response was achieved. The resulting gold electrode was then subjected to CV scans in 2.0 mM K_4_Fe(CN)_6_ + 0.1 M Na_2_SO_4_ solution (−0.1 to 0.5 V, 50 mV s^−1^). A peak-to-peak potential width of ca. 70 mV indicated that the electrode had been properly cleaned.

The gold electrode was placed in a 3.0 mM H_2_PtCl_6_ + 0.1 M HClO_4_ solution and electroplated at a constant potential of 0 V for 300 s to obtain a Pt-modified Au electrode (Pt_pla_/Au). The obtained Pt_pla_/Au electrode was placed in 0.1 M H_2_SO_4_ for CV characterization (−0.3–1.5 V, 50 mV s^−1^). After Pt plating, a reduction peak of Pt oxide appeared at 0.35 V, and the adsorption/desorption peak of H on the Pt surface appeared at 0–0.28 V. The reduction peak of Au oxide at 0.85 V nearly disappeared, indicating that the Au electrode surface was fully covered with Pt (Figure S3). Then, using Pt_pla_/Au as the working electrode, the electrochemical deposition behavior of Pb on the Pt_pla_/Au surface was investigated by the CV method in 3.0 mM Pb(ClO_4_)_2_ + 0.1 M HClO_4_ solution. Similarly, a bare gold electrode was used as the working electrode to study the electrochemical deposition behavior of Pb on the gold electrode surface.

### 3.4. Electrochemical Characterization of the Modified Electrodes and Electrocatalytic Oxidation of Methanol

The modified electrode was subjected to CV characterization in 1.0 M KOH (−1.0 to 0.5 V, 50 mV s^−1^). The electrocatalytic oxidation of methanol was conducted in 1.0 M CH_3_OH + 1.0 M KOH solution (−1.0 to 0.5 V, 50 mV s^−1^). The stability of the modified electrode toward methanol electrocatalytic oxidation was evaluated using a potentiostatic method in 1.0 M CH_3_OH + 1.0 M KOH solution at a potential of −0.25 V for 1800 s. The electrochemical active area of Pt was calculated from the hydrogen adsorption peak in 1.0 M KOH solution, using a conversion factor of 210 μC cm^−2^ [21]. All solutions were deoxygenated with high-purity nitrogen for at least 10 min.

## 4. Conclusions

In summary, a Pb-containing PtAu nanoflower electrocatalyst was deposited on the MWCNT/GCE cathode in a DCGC device. The electrochemical experimental results demonstrate that the bulk dissolution of Cu at the anode can induce the UPD of Pb on Pt at the cathode during the GRR process in the DCGC device. The UPD effect of Pb influenced the growth process of PtAu nanoparticles, leading to the formation of Pb-containing PtAu nanoflower structures. Under the optimal experimental conditions, the prepared Pb-Pt3Au1/MWCNTs/GCE showed the highest activity and stability for the electrocatalytic oxidation of methanol in an alkaline environment. The method of depositing high-activity electrocatalysts via GRR in the DCGC device is expected to have broad application value in electrocatalysis and fuel cell research.

## Figures and Tables

**Table 1:** Abbreviation and the corresponding metal salt composition of catholyte for preparing the modified electrodes.

Abbreviation of Modified Electrodes	The Metal Salt Composition of Catholyte
Pb-Pt/MWCNTs/GCE	3.0 mM H_2_PtCl_6_ + 5.0 mM Pb(ClO_4_)_2_ + 0.1 M HClO_4_
Pb-Pt_3_Au_0.5_/MWCNTs/GCE	5.0 mM Pb(ClO_4_)_2_ + 3.0 mM H_2_PtCl_6_ + 0.5 mM HAuCl_4_ + 0.1 M HClO_4_
Pb-Pt_3_Au_1_/MWCNTs/GCE	5.0 mM Pb(ClO_4_)_2_ + 3.0 mM H_2_PtCl_6_ + 1.0 mM HAuCl_4_ + 0.1 M HClO_4_
Pb-Pt_3_Au_3_/MWCNTs/GCE	5.0 mM Pb(ClO_4_)_2_ + 3.0 mM H_2_PtCl_6_ + 3.0 mM HAuCl_4_ + 0.1 M HClO_4_
Pb-Pt_3_Au_5_/MWCNTs/GCE	5.0 mM Pb(ClO_4_)_2_ + 3.0 mM H_2_PtCl_6_ + 5.0 mM HAuCl_4_ + 0.1 M HClO_4_
Pt_3_Au_1_/MWCNTs/GCE	3.0 mM H_2_PtCl_6_ + 1.0 mM HAuCl_4_ + 0.1 M HClO_4_
Pb-Au/MWCNTs/GCE	3.0 mM HAuCl_4_ + 5.0 mM Pb(ClO_4_)_2_ + 0.1 M HClO_4_

## Data Availability

Data are contained within the article and Supplementary Materials.

## Supplementary Materials

The following supporting information can be downloaded at: https://www.mdpi.com/article/10.3390/molecules29235492/s1. Figure S1. XRD patterns of the as-synthesized Pt_3_Au_1_ and Pb-Pt_3_Au_1_ sample. Figure S2. XPS spectra of the Au (4f), Pt 4f (a) and Pb 4f (b) regions for the as-synthesized Pt_3_Au_1_ and Pb-Pt_3_Au_1_ sample. Figure S3. CV curves at Pt_pla_/Au and bare Au electrodes in 0.1 M H_2_SO_4_ aqueous solution. Scan rate: 50 mV s^−1^.
