# Supplementary material for: Fabrication of Pb-Containing PtAu Nanoflowers via Galvanic Replacement Method for Electrocatalytical Oxidation of Methanol
Source: Molecules. 2024 Nov 21;29(23):5492. doi: 10.3390/molecules29235492 (PMC11643685; doi:10.3390/molecules29235492)
Supplement: Supplementary file 1 [file molecules-29-05492-s001.zip › molecules-3218084-supplementary.pdf]

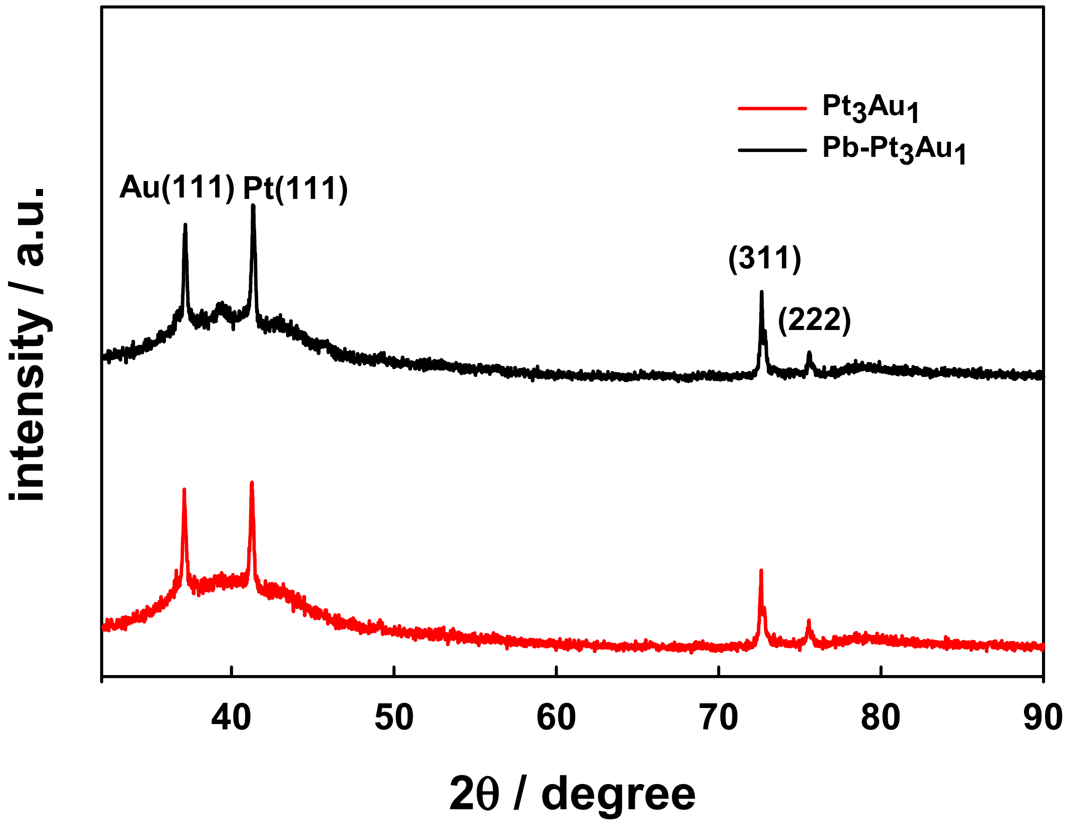

Figure S1. XRD patterns of the as-synthesized Pt_3Au_1 and Pb-Pt_3Au_1 sample.

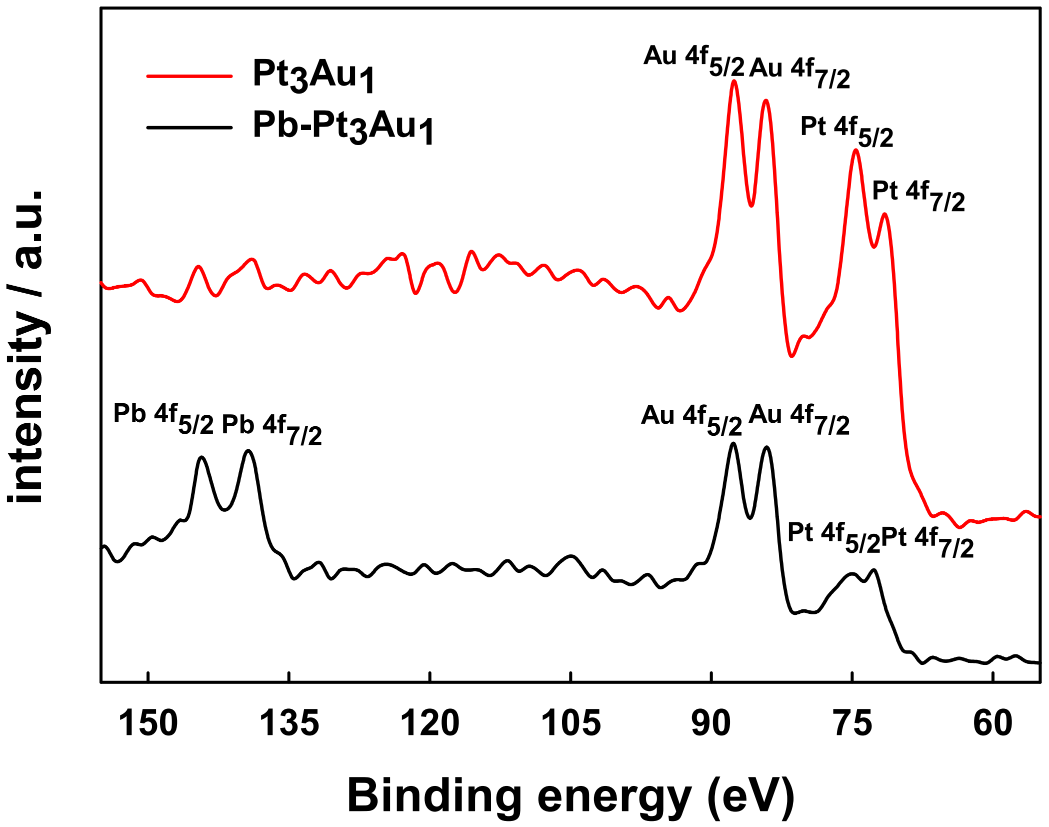

Figure S2. XPS spectra of the Au (4f), Pt 4f (a) and Pb 4f (b) regions for the as-synthesized Pt_3Au_1 and Pb-Pt_3Au_1 sample.

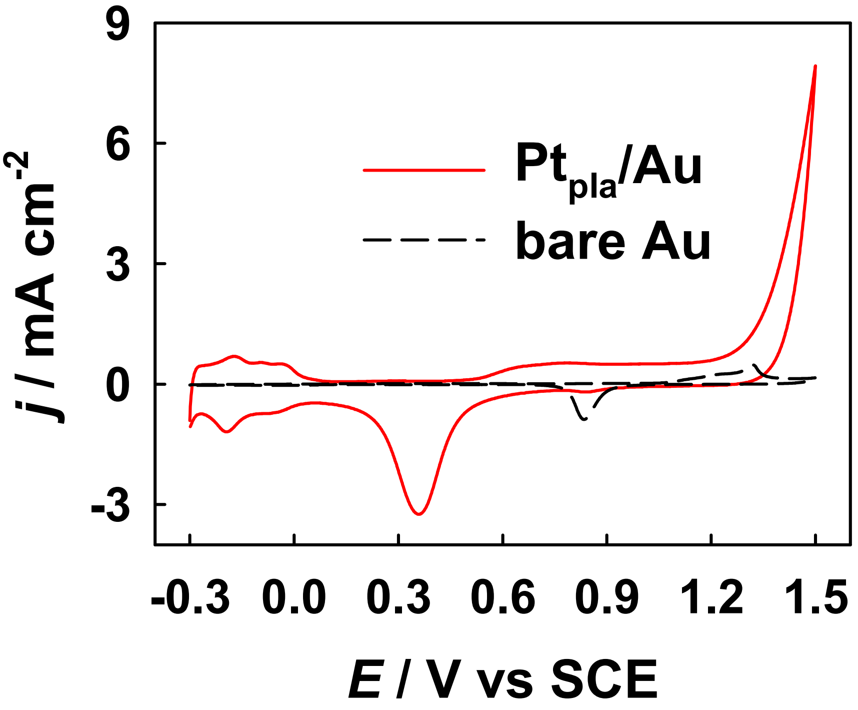

Figure S3. CV curves at Pt_{pla}/Au and bare Au electrodes in 0.1 M H_2SO_4 aqueous solution. Scan rate: 50 mV s^{-1}.
